# Indicators of recent COVID-19 infection status: findings from a large occupational cohort of staff and postgraduate research students from a UK university

**DOI:** 10.1186/s12889-022-13889-0

**Published:** 2022-08-09

**Authors:** Katrina A. S. Davis, Ewan Carr, Daniel Leightley, Valentina Vitiello, Gabriella Bergin-Cartwright, Grace Lavelle, Alice Wickersham, Michael H. Malim, Carolin Oetzmann, Catherine Polling, Sharon A. M. Stevelink, Reza Razavi, Matthew Hotopf

**Affiliations:** 1 King’s College London Institute of Psychiatry Psychology and Neuroscience, London, UK; 2 South London and Maudsley NHS Foundation Trust, London, UK; 3 School of Biomedical Engineering and Imaging Sciences, Faculty of Life Sciences and Medicine, King’s College London, London, UK; 4 Faculty of Life Sciences and Medicine, King’s College London School of Immunology & Microbial Sciences, London, UK

**Keywords:** COVID-19, Cohort studies, Public health, Classification, COVID-19 serological testing

## Abstract

**Background:**

Researchers conducting cohort studies may wish to investigate the effect of episodes of COVID-19 illness on participants. A definitive diagnosis of COVID-19 is not always available, so studies have to rely on proxy indicators. This paper seeks to contribute evidence that may assist the use and interpretation of these COVID-indicators.

**Methods:**

We described five potential COVID-indicators: self-reported core symptoms, a symptom algorithm; self-reported suspicion of COVID-19; self-reported external results; and home antibody testing based on a 'lateral flow' antibody (IgG/IgM) test cassette. Included were staff and postgraduate research students at a large London university who volunteered for the study and were living in the UK in June 2020. Excluded were those who did not return a valid antibody test result. We provide descriptive statistics of prevalence and overlap of the five indicators.

**Results:**

Core symptoms were the most common COVID-indicator (770/1882 participants positive, 41%), followed by suspicion of COVID-19 (*n* = 509/1882, 27%), a positive symptom algorithm (*n* = 298/1882, 16%), study antibody lateral flow positive (*n* = 124/1882, 7%) and a positive external test result (*n* = 39/1882, 2%), thus a 20-fold difference between least and most common. Meeting any one indicator increased the likelihood of all others, with concordance between 65 and 94%. Report of a low suspicion of having had COVID-19 predicted a negative antibody test in 98%, but positive suspicion predicted a positive antibody test in only 20%. Those who reported previous external antibody tests were more likely to have received a positive result from the external test (24%) than the study test (15%).

**Conclusions:**

Our results support the use of proxy indicators of past COVID-19, with the caveat that none is perfect. Differences from previous antibody studies, most significantly in lower proportions of participants positive for antibodies, may be partly due to a decline in antibody detection over time. Subsequent to our study, vaccination may have further complicated the interpretation of COVID-indicators, only strengthening the need to critically evaluate what criteria should be used to define COVID-19 cases when designing studies and interpreting study results.

**Supplementary Information:**

The online version contains supplementary material available at 10.1186/s12889-022-13889-0.

## Introduction

The vast majority of people who experience a COVID-19 illness will not require hospitalisation for that illness but stay in the community [1–3]. Proportions of people requiring hospitalisation vary over time and place, and rely on getting an accurate incidence of COVID-19, but one modelling study estimated that only 2% of people with COVID-19 were admitted to hospital in the first wave of COVID-19 in the UK [4]. Research is urgently needed about medium and long-term outcomes of cases outside the hospital, particularly so-called "long COVID" [5–10]. In hospitals, COVID-19 status is determined using clinical assessment and investigations, particularly antigen detection by polymerase chain reaction (PCR) on nasopharyngeal swab samples and lung imaging (usually Computed Tomography, CT); therefore, hospital-based cohorts can have a strong basis for COVID-19 diagnosis [11]. In community settings, particularly during the first wave of COVID-19, such information was often unavailable, [4] and may continue to be so in resource-limitedenvironments. As investigations for COVID-19 are time-sensitive, participants in cohort studies may have missed the window where definitive diagnosis might be made. Since there are no "gold standard" methods by which community-based studies can distinguish between cases and controls, researchers have had to rely on proxy indicators of COVID-19. Despite guidance on clinical tests for COVID-19, [11–15] there is little evidence aimed explicitly at choosing and interpreting proxy indicators of past COVID-19 infection in research contexts.

Potential indicators of past COVID-19 infection (COVID-indicators) include self-report – for instance, an individual’s belief they have had COVID-19, symptoms they recall and test results they report. These are all dependent on participant recall. Some variables may also be available through electronic health records, that are not prone to recall effects, but bias case-finding towards those in contact with health services during the pandemic [16, 17]. To provide an objective measure for past infection, it is possible to detect antibodies to SARS-CoV-2 (the virus that causes COVID-19) in blood samples, but it is not yet clear how long these antibodies remain detectable [18]. This paper describes concordance between proxy COVID-indicators, both self-report and antibody, in a cohort study of staff and postgraduate research students (PGRs) of a university in London, United Kingdom (UK) [19] during the first wave of the pandemic (winter-spring 2020). The aim is to provide evidence to inform the design and the interpretation of future studies of COVID-19 in non-hospitalised participants.

## Methods

This cohort study conforms to The Strengthening the Reporting of Observational Studies in Epidemiology (STROBE) reporting guidelines, [20] documented in appendix 1 of supplementary materials. Ethical approval was granted by King's Psychiatry, Nursing and Midwifery Research Ethics Committee (HR-19/20–18,247) and research was performed in accordance with the Declaration of Helsinki.

### Setting

The King's College London Coronavirus Health and Experiences of Colleagues at King's (KCL CHECK) study explores the health and wellbeing outcomes of the COVID-19 pandemic on staff and PGRs. A protocol is available [19]. Briefly, on 16 April 2020, all KCL staff and PGRs were invited via email and internal social media to participate in an online survey (‘the baseline survey’). The survey was open for enrolment for two weeks. Participants provided informed consent for their data to be used internally and for research purposes, and were given the opportunity to opt in to follow-up surveys (surveys every two months and shorter surveys every two weeks) and antibody testing.

### Participants

All KCL CHECK participants who consented to follow-up and gave a valid UK address were sent a test kit. Participants residing outside the UK in June 2020 were excluded for logistical reasons. Participants were included in this analysis if they returned a valid antibody test result by 13^th^ July 2020.

### Data collection

Table 1 shows the schedule for follow-up surveys, with the first referred to as Period 1 (P1). Questions in the baseline and longer follow-up surveys asked about experiences in the last two months (e.g. P4); questions in the shorter fortnightly surveys referred to the last two weeks. This analysis reports data from surveys at P0 (baseline) to P5, which took place between April and June 2020.

**Table 1:** Periods of data collection for KCL CHECK to week 18 (April – Aug 2020)

	*Timepoint*								
Data collection period	P0 (Baseline)	P1	P2	P3	P4	P5	P6	P7	P8
*Week of study*	*1–3*	*3–4*	*5–6*	*7–8*	*9–10*	*11–12*	*13–14*	*15–16*	*17–18*
*Month*	*April*	*May*		*June*			*July*		*Aug*
Long survey • "In the last two months"	**x**				**x**				**x**
*Short survey* • "In the last two weeks"		**x**	**x**	**x**		**x**	**x**	**x**	
*Antibody test*						**x**			

The antibody test provided was the SureScreen Diagnostics Rapid COVID-19 IgG/IgM Immunoassay Test Cassette, which detects antibodies to the 'spike' protein of SARS-CoV-2. The performance of this test (under laboratory conditions) has been shown to be good. For example, using samples from 268 keyworkers who self-reported positive COVID-19 antigen/PCR tests and 1,995 historical samples, it had 94.0% sensitivity and 97.0% specificity, also showing 96.3% agreement with the SAR-CoV-2 spike antibody enzyme-linked immunoassay (ELISA) result for 2,847 keyworkers [21]. An internal pilot demonstrated that participants could use the test cassette without specific training [22]. We developed our procedure and detailed illustrated instructions following pilot feedback, shown in supplementary material appendix 2. In late June 2020, the test kit was posted to participants, including the test cassette and a lancet for providing a blood spot. Participants uploaded a photograph of their result to a secure server. Participants were asked to email the team if they had difficulties, who answered within two working days and could arrange for a replacement kit (sent in early July 2020).

### Deriving COVID-indicators

Self-reported COVID-indicators were measured at baseline and follow-up surveys as follows.

- Suspicion of COVID-19 illness: At baseline (P0), participants were asked, "Do you think that you have had COVID-19 (coronavirus) at any time? Definitely/Probably/Unsure/No". At P1, P2, P3, and P5, participants were asked, "Do you think that you have had COVID-19 (coronavirus) in the last two weeks?" At P4, participants were asked, "Do you think that you have had COVID-19 (coronavirus) in the last two months?". Positive suspicion was defined as a response of "Definite" or "Probable" in any survey (P0-P5).

- COVID-19 symptoms: We used a symptom list derived from the ZOE coronavirus daily reporting app (part of the COVID symptom study, [23, 24]), adapted to cover two-month periods (P0 and P4) or two-week periods (P1, P2, P3 and P5) and used to define multiple possible symptom states. (a) Any symptom: Responded to the screening question "How have you felt physically?" with 'Not quite right' rather than 'Normal' (b) Core symptoms: Any report of 'fever', 'new persistent cough' or 'loss of smell/taste'. (c) Symptom algorithm: The COVID symptom study reported an algorithm (including age, gender, core symptoms of COVID-19, 'severe fatigue' and 'skipped meals') with scores above a cut-off representing a high likelihood to have COVID [23]. Combining these definitions (a-c), a positive algorithm was considered the most specific category, followed by core symptoms and then any symptoms. An overall symptom category was assigned as the most specific category reached in any survey (P0-P5).

- COVID-19 test results. We asked, "Have you had a test for COVID-19 (coronavirus)?" and "What was the result?" at baseline, repeated at P1, P2, P3, and P5 for the two preceding weeks and at P4 for the preceding two months. We did not ask for any evidence. Those who reported a positive test at any point P0-P5 were defined as positive on external tests. To further differentiate between tests for current infection and tests for previous infection, at P8, we asked about different types of tests. Those who endorsed having a "blood/blood spot test to look for evidence of past infection" (excluding the test they had received through the KCL CHECK study) were allocated 'external antibody test'. Those who had reported a test in P0-P5 and endorsed "swab of the throat and/or nose to look for infection" or who did not report at P8 were allocated 'antigen/PCR test'.

Because each indicator is summarised over time as positive if ever met and negative in all other cases, missing values caused by participants not completing a survey were treated as a negative result at that time.

Antibody result: The results of KCL CHECK home antibody testing for spike IgM or IgG using the SureScreen Diagnostics Rapid COVID-19 Immunoassay Test Cassette results were adjudicated by the KCL CHECK team based on an uploaded photograph, as explained in a previous paper [22] and illustrated in supplementary material appendix 2. Extraction of these results took place on 13 July and included all photos uploaded by this date.

### Participant characteristics

All characteristics were self-reported in the baseline survey. Ethnicity was asked using recommended wording from the Office of National Statistics with 18 groups, [24] reported grouped into categories due to small numbers of some of the 18 ethnic groups. Participants were also asked whether they were “key workers” based on government definitions of essential workers. Population characteristics of all staff and PGRs students at King’s College London were obtained from KCL administrative sources to describe sample representativeness.

### Analysis

Datasets from each period (P0-5 and P8) and antibody testing were merged using R 4.0.0 and associated packages [25–28]. We summarised participation and missing data. Overlap of the indicators was explored through descriptive analyses and figures. *Concordance* between pairs of indicators is the proportion of participants in whom both indicators agree (both positive or both negative). Additionally, we compared self-report indicators to the KCL CHECK antibody tests using sensitivity (proportion of antibody positive participants also identified by indicator) and specificity (proportion of antibody negative participants also negative by indicator). However, the use of these statistics does not imply that we regard the antibody test results as a gold-standard for defining past COVID-19 cases. We give proportions to the nearest percentage point, unless under 1%, with 95% confidence intervals calculated using Wilson's method.

## Results

### Participants

**Fig. 1:**
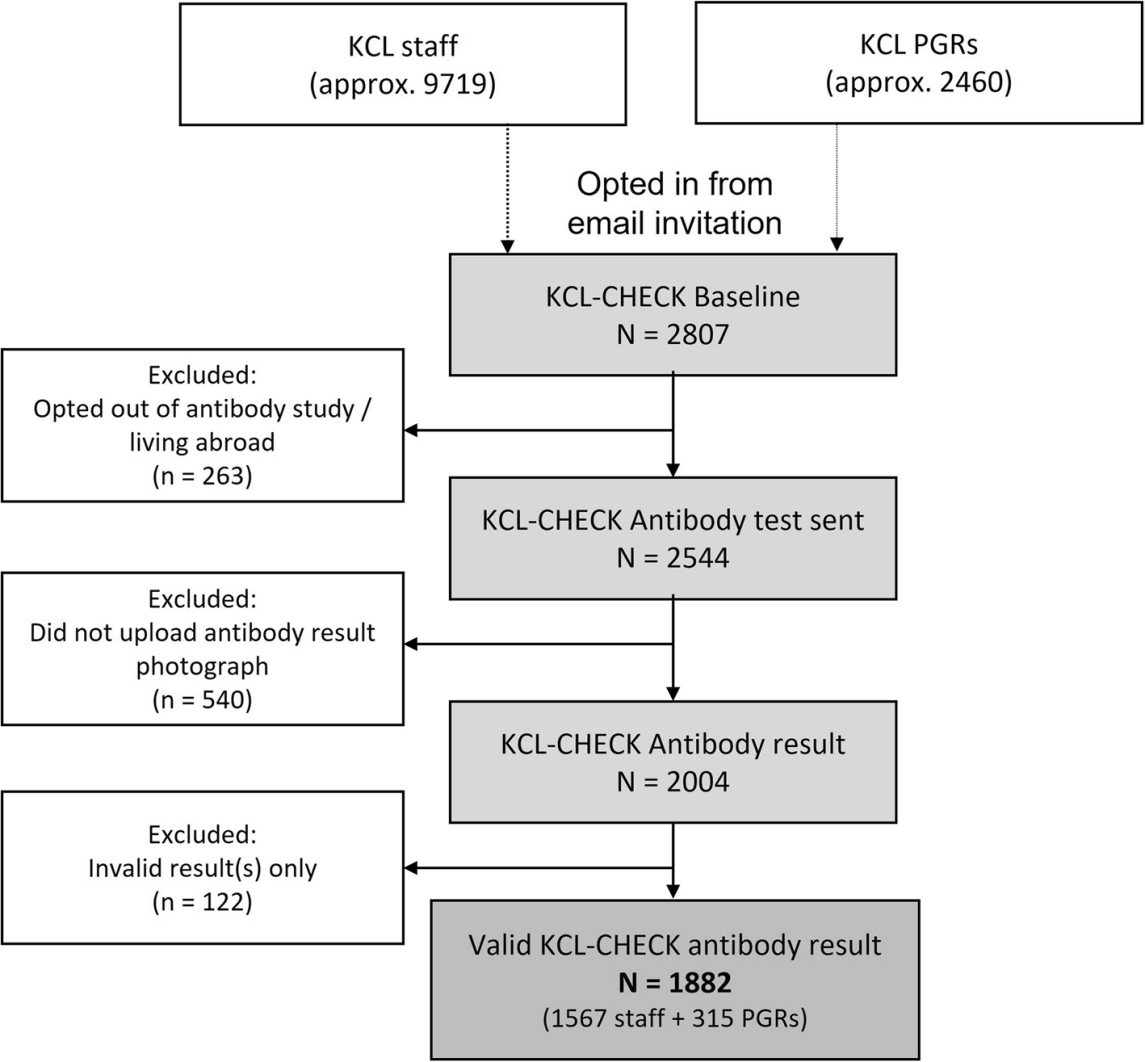
Study flowchart

Out of approximately 9719 staff and 2460 PGRs in KCL, 2807 (23%) volunteered for KCL CHECK (see Fig. 1). A total of 2544 participants who consented to the longitudinal study and antibody testing, and who were residing in the UK, received a testing kit. Excluding those who did not return a valid result, left 1882 participants for analysis. Table ST1 in supplementary materials shows these exclusions did not greatly affect the composition of the sample.

### Sample characteristics

In the analytical sample (*n* = 1882) 88% identified as being from a White ethnic background, 71% identified as female and 13% as keyworkers, with a median age of 37 years. Compared to the characteristics of the university staff (Table ST2 in supplementary materials) and PGRs (Table ST3 in supplementary materials), White and female people were over-represented in our sample.

The antibody test was sent to participants 10–12 weeks after they completed their baseline survey. Table 1 shows five opportunities to complete follow-up surveys in this time. 98% of the sample completed at least one survey, and 68% completed all five. 1687 (90%) participants took part in the first two-month survey (P4), which comprised eight of the 10–12 weeks that elapsed. We considered these in a secondary analysis to see whether more complete reporting would alter the results. Prevalence and overlap of COVID-indicators were identical in the cohorts, so the larger cohort is reported.

### Prevalence of COVID-indicators

Table 2 shows the prevalence of COVID- indicators in our sample. Of 1882 participants, 124/1882 (7%, 95% confidence interval 6–8%) tested positive on the study antibody test. Core symptoms were reported by 770/1882 (41%, CI 39–43%), 90% (694/770) of these experienced those symptoms before the baseline survey (April 2020). 298/1882 (16%, CI 14–18%) also met the criteria for the symptom algorithm. Suspicion of having had COVID (probable or definite) was reported by 509/1882 (27%, CI 25–29%), 96% (487/509) experienced this before baseline. 323 participants reported they had been tested elsewhere (235 antigen test only, 138 antibody test only, 50 both) with 39/1882 (2%, CI 2–3%) reporting at least one positive external test result (six antigen positive, 29 antibody positive, four both). This means that 10 of our 1882 participants (0.5%, CI 0.3–1%) is known to have had a positive antigen test during this wave of COVID-19.

**Table 2:** Prevalence and overlap of positive COVID-19 indicators in KCL CHECK (*n* = 1882)

	One or more core COVID-19 symptoms reported	Participant thinks they have had COVID-19	Symptom algorithm positive	KCL CHECK antibody test positive	Reports positive test result from elsewhere
Overall prevalence	770/1882, 41%	509/1882, 27%	298/1882, 16%	124/1882, 7%	39/1882, 2%
Number and proportion of column who also have:					
One or more core COVID symptoms reported		429 / 509, 84%	298 / 298, 100%	106 / 124, 85%	31 / 39, 79%
Participant thinks they have had COVID	429 / 770, 56%		214 / 298, 72%	101 / 124, 81%	33 / 39, 85%
Symptom algorithm positive	298 / 770, 39%	214 / 509, 42%		83 / 124, 67%	25 / 39, 64%
KCL -CHECK antibody test positive	106 / 770, 14%	101 / 509, 20%	83 / 298, 28%		24 / 39, 62%
Reports positive test result from elsewhere	31 / 770, 4%	33 / 509, 6%	25 / 298, 8%	24 / 124, 19%	

Indicators in order of prevalence in the main cohort

When gender, age and ethnicity are considered (see Table ST4 in supplementary materials), proportions positive on the study antibody test were broadly the same in all groups. However, younger age groups reported core symptoms more often (44% in under 45 s, 34% in 45 +) and men reported suspicion of COVID-19 illness more often (33% in men, 25% in women). There were no significant results by ethnicity, but given small numbers there was low certainty around the estimates for Asian and other minority ethnic groups.

### Overlap between alternative indicators of COVID-19

We found overlap between alternative indicators (Table 2) whereby any indicator being positive increased the likelihood of other indicators being positive. For instance, those with core symptoms had double the proportion of positive tests – both study tests (106/770, 14%) and external (31/770, 4%) – compared to those without core symptoms. Of those who tested positive on the KCL CHECK antibody tests, 85% had experienced core symptoms, 81% thought they had had COVID-19, and 67% met the symptom algorithm. In the KCL CHECK antibody-positive group, 28% reported receiving an external test, and 19% reported at least one positive external test. Concordance of indicator status between pairs of outcomes is shown in supplementary material table ST5. Concordance ranged from 60% for core symptoms and external test to 94% for the KCL CHECK antibody test and external test.

**Fig. 2:**
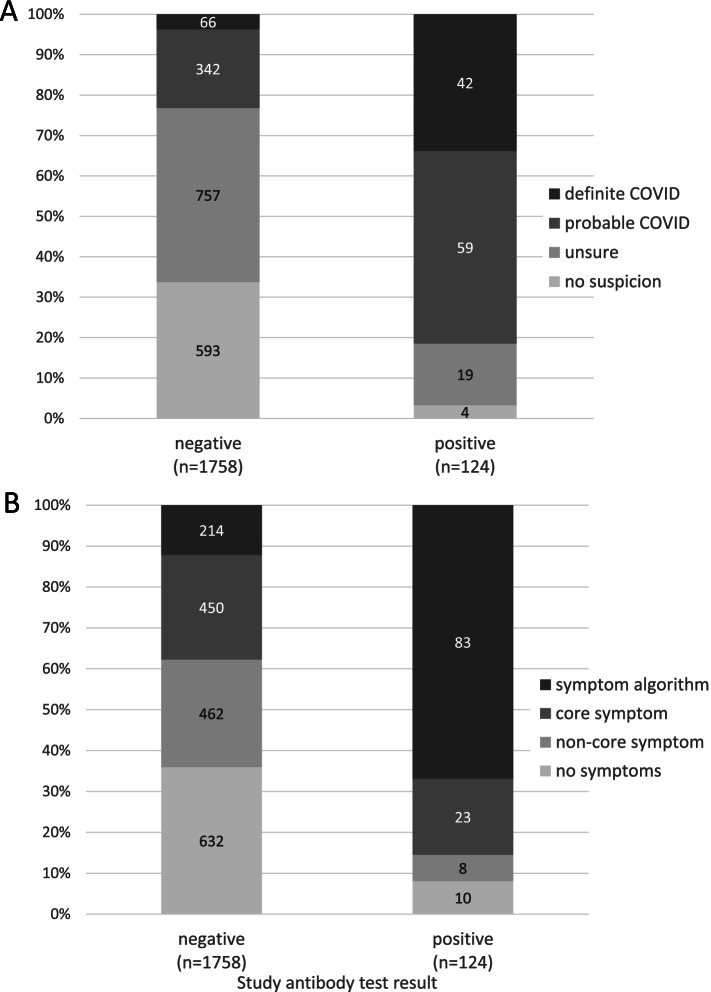
(**A**-**B**) KCL CHECK antibody test result in June by suspicion and symptoms. **A**. participant suspicion that they had experienced COVID-19; **B**. highest level of symptoms

Figure 2A and Table ST6 show that participants who thought they had not experienced COVID-19 were very unlikely to get a positive antibody test result (4/597, 0.7%). Probable or definite suspicion of COVID-19 infection had 81% sensitivity (101/124) and 77% specificity (1350/1758) for the KCL CHECK antibody test. Figure 2B and Table ST7 show that most people who tested positive on the KCL CHECK antibody test were positive on the symptom algorithm, which had 67% sensitivity (83/124) and 88% specificity (1544/1758) for the KCL CHECK antibody test. Core symptoms (including those also algorithm positive) had 85% sensitivity (106/124) and 62% specificity (1094/1758).

Combining the range of symptom and suspicion reports, ST8 shows the number of participants at each intersection of symptom and suspicion level. Adding KCL CHECK antibody test results, Table 3 and Table ST9 show the proportion testing positive in each intersect (except where there were fewer than ten participants). Table 3 shows that, within those of the same symptom level, greater suspicion of having had COVID-19 also had a greater proportion antibody positive. Of those who were positive for the symptom algorithm and had definite suspicion, 49% were positive on the KCL CHECK antibody test.

**Table 3:** Intersect of suspicion and self-reported symptoms domains, showing proportion of KCL CHECK antibody test for participants in each intersect^a^

		% KCL CHECK antibody positive				
		Highest suspicion reported				
		no suspicion (*n* = 597)	unsure (*n* = 776)	probable (*n* = 401)	definite (*n* = 108)	Totals
Most specific symptoms reported	symptom algorithm (*n* = 298)	NR	13%	27%	49%	**28%**
	core symptoms (*n* = 472)	0%	1%	8%	17%	**5%**
	non-core symptoms (*n* = 470)	0%	2%	6%	NR	**2%**
	no symptoms (*n* = 642)	1%	2%	4%	NR	**2%**
	Totals	**1%**	**2%**	**15%**	**39%**	**7%**

*NR* Not reported, as less than 10 participants in intersecting cell

^a^See supplementary material tables ST8 and ST9 for more detail

### Secondary analyses

Table ST10 compares the external antibody test results and KCL CHECK antibody test results for the 138 participants with external antibody test results. Concordance was 88%. Using the external tests would indicate 24% (CI 18–32) of the 138 participants positive for antibodies, but this falls to 15% (CI 10–22) positive on the KCL CHECK antibody test.

## Discussion

Ascertaining cases and controls of COVID-19 in the community is challenging. [1] There are no ‘gold-standard’ diagnostic criteria, [11] and for people who have not had the time-sensitive tests, there may be no opportunity to get diagnostic certainty. KCL CHECK used retrospective ascertainment of COVID-19 based on multiple COVID-indicators. Self report COVID-indicators included suspicion of COVID-19, symptoms and test results. An objective measure was home antibody testing. The results show that depending on which COVID-indicator is used, there could be a wide range of estimates of COVID-19 illness history. Our cohort had prevalence of COVID-indicators that ranged from 2% (external test positive) to 41% (core symptom of COVID) in June 2020. Self-reported positive external tests were reported by the fewest participants – but given that antigen testing in the community was unavailable during the March 2020 wave of infections (only 285 (15%) of the cohort had accessed antigen testing by June 2020) this will greatly under-estimate the prevalence of mild-moderate cases of COVID-19. Antigen testing (both PCR and lateral-flow) in the UK expanded subsequently, but performance of the test depends on timing and swab technique, [14] while accessing tests and reporting results requires engagement with authorities, [29] such that reporting of positives from routine antigen tests probably still underestimates true COVID-19 prevalence [30]. Reporting a core COVID-19 symptom, in contrast, is likely to over-estimate true prevalence of COVID-19 since the symptoms overlap with other common illnesses. Other indicators were suspicion of having had COVID-19 (reported by 25% of female and 33% of male participants), symptom algorithm (positive for 16%) and antibody testing (positive for 7%).

### Antibody testing

Antibody testing in KCL CHECK used an IgG/IgM test kit based on “lateral flow” technology, sent to participants, which was simple to use and has high validity (under lab conditions) [31]. Antibody tests can give false positives in up to 2 per 100 tests, through cross-reactivity with antibodies unrelated to SARS-CoV-2, [12, 21] which can be problematic in large studies with low prevalence. Testing negative after having had COVID-19 is also a concern, since small numbers of people do not produce anti-spike antibodies, [32, 33] they are detected more inconsistently in mild and asymptomatic COVID-19, [31, 34, 35] and decline over time [36–38]. Testing in KCL CHECK (June-July 2020) occurred at least three months after the onset of most participants’ symptoms (Feb-March 2020). Antibodies may cease to be detectible some months after exposure, especially on lateral flow devices [39, 40]. We found 7% of our participants were positive for anti-spike IgG/IgM. Comparing to two studies done at around this time in England, the proportion of positive antibody results is lower than in TwinsUK (12%) [41] and around the level of REACT-2 (6%) [42]. However, these findings are not directly comparable, as TwinsUK used ELISA (which is more sensitive than lateral flow tests) and REACT-2 had a different participant profile. Among KCL CHECK participants who reported previous antibody testing, 15% were positive on the KCL CHECK antibody test, compared with 24% in their prior reported test. This may suggest time-dependent loss of reactivity, although there were likely also differences in test specifications and this comparison is based on small numbers of participants. Further rounds of testing of our cohort may help clarify this [43]. Augmenting antibody testing with testing for T cell response to better track long-term immunity may be possible in the future [44].

### Agreement between antibody testing and other indicators

Some other studies have explored the agreement of symptoms and objective test results. The COVID symptom study found that their algorithm had high sensitivity (65%) and specificity (78%) for antigen/PCR test results [23]. Researchers compared the algorithm with antibody testing in the TwinsUK cohort, where being algorithm positive in daily symptom recording at any point in March to April was 37% sensitive and 95% specific for antibodies via ELISA in April-June [41]. In our study, the algorithm was more sensitive (67%) and slightly less specific (88%), indicating that a greater proportion of people who were antibody positive had significant symptoms. The known epidemiology for COVID-19 suggests that a proportion of people have COVID-19 without any obvious symptoms, [1] and thus a proportion of those who were antibody positive would not recall symptoms. In our study, 10 people with no symptoms tested positive (10/632, 1.6%), as well as 8 people who reported feeling "not quite right" but did not have core symptoms (8/462, 1.7%), making up 15% (18/124) of those who were antibody positive. This is low compared to the proportion without core symptoms who tested positive in TwinsUK (27%) and REACT-2 (39%) [41, 42].

We found that for a given level of symptoms, a higher suspicion of having had COVID-19 added to the likelihood of testing positive; presumably because a participant's suspicion includes context such as symptom unusualness and contacts with COVID-19. However, it is also true that even for participants who were definite they had experienced COVID-19 and had symptoms severe enough to be positive on the symptom algorithm, the majority (51%) were negative on antibody testing. This surprisingly low level of confirmation, taken together with other studies that have tested of our lateral flow device [31, 32] and comparison with TwinsUK and REACT-2, [45] suggests that the home antibody testing was of low sensitivity –missing some past cases of COVID-19 that may have been positive if subject to laboratory testing or testing closer to the time of illness [45].

### Implications

There was a 20-fold difference in apparent prevalence of past COVID-19 between different COVID-indicators, which means that it is important to consider how a history of COVID-19 has been ascertained when interpreting studies that report past COVID-19 illness. In particular, our results suggest that cohort studies using external testing (which will include record-linkage to testing results, e.g. in UK Biobank [17]) will underestimate the proportion with COVID-19. The under-ascertainment may not be evenly seen across characteristics of the population or illness (e.g. people who had more severe symptoms will have been more likely to be tested), and that can incorporate bias or lead to a lack of generalisability of results [46]. While this is most relevant for infections in the first wave when testing in the UK was severely limited, accessibility of testing may be limited and likely skewed at times of greatest infection and in places with poorer infrastructure or where there are low levels of trust in the authorities [29]. Also notable is that while antibody testing gives an objective outcome, timing is important, and accuracy may be an issue depending on how it was used; in our study likely leading to an under-estimate of cases.

For researchers designing studies and considering which COVID-indicator to use, multiple measures may be preferable. As the difficulties in retrospective ascertainment mean there is underlying uncertainty, a small number of compound variables (e.g., one wide definition and one narrow definition) may be more suitable than a binary case and control adjudication. More specifically, collecting symptom report for the COVID symptom study algorithm [23] will assist in finding those who have had a typical COVID-19-like illness, and considering the participants' own suspicion can add both sensitivity and specificity. Adding a high specificity antibody test to self-report may help to identify past cases that were asymptomatic or atypically symptomatic. However, the low sensitivity of tests such as ours means that they are unlikely to make a good 'rule-out' for people who suspect they had COVID-19 some months before. More generally, there are great benefits to be had from a standardised set of criteria that can be used across studies for reproducibility and comparison, [47] but this does not need to be at the expense of a plurality of approaches that are tailored to the aims of each study.

### Strengths and weaknesses

The strengths of this study include the survey repeating every fortnight to minimise recall bias. We incorporated a symptom checklist that has been previously evaluated. The antibody test kit was highly specific for SARS-CoV-2, suited to minimise false positives in population screening. Home testing maximised uptake of the test at a time when people may have been hesitant about attending a clinic. We attempted to reduce inter-individual variation through our pilot, illustrated instructions and responsive email enquiry team [22].

There are three key limitations of which to be aware. The first regards the antibody test lateral flow cassettes, which are designed for use by a trained person rather than the general public, and are known to be less sensitive and more inconsistent when testing outside the laboratory and on capillary blood [21, 35]. Secondly, as we did not exclude people with missing longitudinal survey we had incomplete data about self-reported items after the baseline. However, COVID-19 infections were much less common in May–June 2020 than they had been in March, [48] so we would expect relatively few positives to occur after the April baseline. This, and a comparison involving participants with more complete data, leads us to believe that this had minimal effect on our outcomes. Thirdly, our cohort was not representative of the general population, and even within our target population (all KCL staff and PGRs) female gender and White ethnicity were overrepresented in our cohort [49].We expect that our general findings will still be useful to studies that have different participant composition, but care would need to be taken in populations with very different expected COVID-19 prevalence. Since the time of this analysis there have been a number of further waves of COVID-19 throughout the world, which may lead to a higher proportion of people having been affected. There has also been a vaccination roll-out nationally, which makes antibody test results much more complicated to interpret.

## Conclusions

This paper compares alternative indicators of past COVID-19 illness and acknowledges the complicated context: the time-course of detectable antigen and antibody, initial poor access to routine testing, symptoms common to other respiratory illnesses, asymptomatic infection, and the high profile of the illness. We found that there was overlap in the occurrence of the indicators, as expected since they reflect similar underlying concepts, but there are large differences in prevalence of different indicators in the community. This analysis is from relatively early in the COVID-19 pandemic, and the prevalence of COVID-indicators will have been affected by events such as vaccination since our analysis. However, our findings are still of relevance for public health planning insofar as they highlight the importance of indicator choice when ascertaining past COVID-19 status, and how the choice of indicator has a large influence on the proportion of a cohort who will be identified as having a history of COVID-19. This may go on to influence downstream results and findings from a cohort. We encourage researchers to consider the use of algorithms that maximise COVID-19 case history, rather than relying on single measures which may give a false sense of certainty.

## Supplementary Information

**Additional file 1.**

## Data Availability

Researchers may access pseudonymised data by upon reasonable request to the Principal Investigators (Professor Matthew Hotopf and Professor Reza Razavi, email: check@kcl.ac.uk).

## Abbreviations

ELISA: Enzyme-linked immunoassay [used for quantitively measuring antibodies]
KCL CHECK: King's College London Coronavirus Health and Experience of Colleagues at King's
P0-P8: Periods in the research study, each with one survey issued
PCR: Polymerase chain reaction [used to detect SARS-CoV-2 antigen]
PGRs: Postgraduate Research Students
