# Supplementary material for: Indicators of recent COVID-19 infection status: findings from a large occupational cohort of staff and postgraduate research students from a UK university
Source: BMC Public Health. 2022 Aug 9;22:1514. doi: 10.1186/s12889-022-13889-0 (PMC9363143; doi:10.1186/s12889-022-13889-0)
Supplement: Supplementary file 1 — Additional file 1. [file 12889_2022_13889_MOESM1_ESM.pdf]

Supplementary Material for paper “Indicators of past COVID-19 infection status: Findings from a large occupational cohort of staff and postgraduate research students from a UK university.” Authors: Katrina A. S. Davis* [1,2], Ewan Carr[3], Daniel Leightley[1], Valentina Vitiello[4], Gabriella Bergin-Cartwright[1.2], Grace Lavelle[1], Alice Wickersham[1,2], Michael H. Malim[5], Carolin Oetzmann[1], Catherine Polling[1.2], Sharon A.M Stevelink[1], Reza Razavi[+ 4], Matthew Hotopf[+ 1,2], On behalf of the KCL CHECK research team

Supplementary Tables (ST1 – ST7)

Appendix 1: STROBE checklist

Appendix 2: Home testing procedure and instructions

Links to supplementary tables

Supplementary Table ST1 Cohort characteristics at different points in the study, with the "valid antibody result" cohort being used in this paper 2

Supplementary Table ST2 Cohort characteristics staff 3

Supplementary Table ST3 Cohort characteristics PGR students 3

Supplementary Table ST4 Positive COVID-indicators split by demographic factors, ... 4

Supplementary Table ST5 Agreement of COVID-19 status (positive vs negative) between pairs of COVID indicators as a proportion of the cohort 5

Supplementary Table ST6 Comparing participant reported **suspicion** with KCL-CHECK antibody testing result in June 2020 6

Supplementary Table ST7 Comparing participant reported **symptoms** with KCL-CHECK antibody testing result in June 2020 6

Supplementary Table ST8 Intersect of suspicion and self-reported symptoms, number of participants in each exclusive combination. 7

Supplementary Table ST9 Positive KCL CHECK antibody test for each intersecting cell of suspicion and symptoms, with heatmap showing relative proportions of positive tests. 7

Supplementary Table ST10 Comparing external antibody testing results with KCL-CHECK antibody test result (in those with external result) 8

Supplementary Table ST1 Cohort characteristics at different points in the study, with the "valid antibody result" cohort being used in this paper

Cohort name (see figure 1)		Baseline	Longitudinal	Antibody result	Valid antibody result
Gender	Female	1948 (69%)	1769 (70%)	1418 (71%)	1339 (71%)
	Male	842 (30%)	760 (30%)	574 (29%)	533 (28%)
	Other	17 (1%)	15 (1%)	<10 (1%)*	<10 (1%)*
Role	Academic, specialist and management.	1075 (38%)	1060 (42%)	866 (43%)	810 (43%)
	Research, clerical and technical	809 (29%)	797 (31%)	661 (33%)	622 (33%)
	Teaching, facilities and clinical	204 (7%)	199 (8%)	142 (7%)	134 (7%)
	Post Graduate Research Students	536 (19%)	452 (18%)	334 (17%)	315 (17%)
	missing	183 (7%)	36 (1%)	<10 (1%)*	<10 (1%)*
Age-group (years)	18-24	136 (5%)	125 (5%)	93 (5%)	90 (5%)
	25-34	1084 (39%)	948 (37%)	732 (37%)	695 (37%)
	35-44	762 (27%)	698 (27%)	566 (28%)	535 (28%)
	45-54	440 (16%)	409 (16%)	316 (16%)	287 (15%)
	55-64	307 (11%)	288 (11%)	239 (12%)	220 (12%)
	65+	78 (3%)	76 (3%)	58 (3%)	55 (3%)
Ethnicity group	White	2152 (77%)	2178 (86%)	1763 (88%)	1660 (88%)
	Mixed	102 (4%)	46 (1.8%)	34 (1.7%)	29 (1.5%)
	Asian	204 (7%)	191 (7.5%)	133 (6.6%)	125 (6.6%)
	Black	39 (1%)	37 (1.5%)	21 (1%)	20 (1.1%)
	Other	70 (2%)	69 (2.7%)	49 (2.4%)	44 (2.3%)
	missing	240 (9%)	23 (1%)	<10 (1%)*	<10 (1%)*
Keyworker status	Keyworker	358 (13%)	357 (14%)	257 (13%)	242 (13%)
	Non-keyworker	2449 (87%)	2187 (86%)	1747 (87%)	1640 (87%)
Total		2807	2544	2004	1882

*True value supressed due to small cell size

Supplementary Table ST2 Cohort characteristics staff

		Approximate sampling frame (from KCL Human Resources)	Staff cohort (valid antibody test)
Gender	Female	5351 (55%)	1110 (71%)
	Male	4368 (45%)	450 (29%)
Age-group	18-24	464 (5%)	40 (3%)
	25-34	3374 (35%)	496 (32%)
	35-44	2755 (28%)	495 (32%)
	45-54	1794 (18%)	276 (18%)
	55-64	1084 (11%)	207 (13%)
	65+	248 (3%)	53 (3%)
Ethnicity group	White	6779 (70%)	1412 (90%)
	Mixed	415 (4%)	23 (1%)
	Asian	1212 (12%)	88 (6%)
	Black	520 (5%)	14 (1%)
	Other	793 (8%)	28 (2%)
Total		9719	1567

Supplementary Table ST3 Cohort characteristics PGR students

		Approximate sampling frame (from KCL Student Office)	PGRs cohort (valid antibody test)
Gender	Female	1390 (57%)	229 (73%)
	Male	1070 (43%)	83 (26%)
Age-group	18-24	690 (28%)	50 (16%)
	25-34	1315 (53%)	199 (63%)
	35+	455 (18%)	66 (21%)
Ethnicity group	White	1454 (59%)	248 (79%)
	Mixed	130 (5%)	<10 (2%*)
	Asian	556 (23%)	37 (12%)
	Black	84 (3%)	<10 (2%*)
	Other	170 (7%)	16 (5%)
Total		2460	315

* True value suppressed due to small cell size

Supplementary Table ST4 Positive COVID-indicators split by demographic factors,

		Numbers and proportions with 95% confidence intervals				
	n.	One or more core COVID-19 symptoms reported	Symptom algorithm positive	Participant thinks they have had COVID-19	Reports positive test result from elsewhere	KCL CHECK antibody test positive
Female	1339	544, 41% (38-43)	215, 16% (14-18)	333, 25% (23-27)*	26, 1.9% (1.3-2.8)	86, 6% (5-8)
Male	533	219, 41% (37-45)	78, 15% (12-18)	174, 33% (29-37)*	13, 2.4% (1.4-4.1)	36, 7% (5-9)
Under 45	1320	580, 44% (41-47)*	218, 17% (15-19)	389, 29% (27-32)*	28, 2.1% (1.5-3.0)	92, 7% (6-8)
45 and over	562	190, 34% (30-38)*	80, 14% (12-17)	120, 21% (18-25)*	11, 2.0% (1.1-3.5)	32, 6% (4-8)
White	1660	674, 41% (38-43)	260, 16% (14-17)	443, 27% (25-29)	34, 2.0% (1.5-2.8)	105, 6% (5-8)
Asian	125	56, 45% (36-54)	24, 19% (13-27)	44, 35% (27-44)	3, 2.4% (0.8-6.8)	9, 7% (4-13)
All other ethnic groups	93	37, 40% (30-50)	13, 14% (8-22)	21, 23% (15-32)	2, 2.2% (0.6-7.5)	10, 11% (6-19)
Overall	1882	770, 41% (39-43)	298, 16% (14-18)	509, 27% (25-29)	39, 2.1% (1.5-2.8)	124, 7% (6-8)

*95% confidence intervals do not overlap

Supplementary Table ST5 Agreement of COVID-19 status (positive vs negative) between pairs of COVID indicators as a proportion of the cohort

Complete cohort (n=1882)	Core symptoms	Participant suspicion of COVID	Symptom algorithm	KCL -CHECK antibody test	External testing
Proportion with positive outcome	41%	27%	16%	7%	2%
Agreement:	% (n agree)	% (n agree)	% (n agree)	% (n agree)	% (n agree)
Core					
Suspicion	78% (1461)				
Algorithm	75% (1409)	80% (1504)			
KCL -CHECK antibody test	64% (1200)	77% (1451)	86% (1626)		
External test	60% (1135)	74% (1400)	85% (1596)	94% (1767)	

*Supplementary Table ST6 Comparing participant reported **suspicion** with KCL-CHECK antibody testing result in June 2020*

Suspicion of COVID-19 illness*	KCL-CHECK antibody **negative**	KCL-CHECK antibody **positive**	%positive	Prevalence ratio, relative to no suspicion
No suspicion (n=597)	593	4	0.7%	ref
Unsure (n=776)	757	19	2.4%	3.7
Probable COVID (n=401)	342	59	14.7%	22.0
Definite COVID (n=108)	66	42	38.9%	58.0
Total (n=1882)	1758	124	6.6%	--

* Highest level of participant suspicion of COVID-19 reported (April-June 2020 surveys)

*Supplementary Table ST7 Comparing participant reported **symptoms** with KCL-CHECK antibody testing result in June 2020*

Symptoms of COVID-19 illness*	KCL-CHECK antibody **negative**	KCL-CHECK antibody **positive**	%positive	Prevalence ratio, relative to no symptoms
No symptom (n=642)	632	10	1.6%	ref
Non-core symptom (n=470)	462	8	1.7%	1.1
Core symptom (n=473)	450	23	4.9%	3.1
Symptom algorithm positive (n=297)	214	83	27.9%	17.9
Total (n=1882)	1758	124	6.6%	--

* Most specific symptoms reported (April-June 2020 surveys)

Supplementary Table ST8 Intersect of suspicion and self-reported symptoms, number of participants in each exclusive combination.

		N. participants in intersect				
		Highest suspicion reported				
		no suspicion	unsure	probable	definite	overall
Most specific symptoms reported	symptom algorithm	7	76	146	68	**298**
	core symptoms	19	239	180	35	**472**
	non-core symptoms	154	263	51	2	**470**
	no symptoms	417	198	24	3	**642**
	overall	**597**	**776**	**401**	**108**	**1882**

Supplementary Table ST9 Positive KCL CHECK antibody test for each intersecting cell of suspicion and symptoms, with heatmap showing relative proportions of positive tests.

		n antibody positive / N participants in intersect<br>% positive in intersect				
		Highest suspicion reported				
		no suspicion	unsure	probable	definite	overall
Most specific symptoms reported	symptom algorithm	NR*	10 / 76, 13%	40 / 146, 27%	33 / 68, 49%	**83 / 298, 28%**
	core symptoms	0 / 19, 0%	2 / 239, 1%	15 / 180, 8%	6 / 35, 17%	**23 / 472, 5%**
	non-core symptoms	0 / 154, 0%	4 / 263, 2%	3 / 51, 6%	NR*	**8 / 470, 2%**
	no symptoms	4 / 417, 1%	3 / 198, 2%	1 / 24, 4%	NR*	**10 / 642, 2%**
	overall	**4 / 597, 1%**	**19 / 776, 2%**	**59 / 401, 15%**	**42 / 108, 39%**	**124 / 1882, 7%**

** Not reported as less than ten participants in intersect cell, but values included in row and column totals.*

Supplementary Table ST10 Comparing external antibody testing results with KCL-CHECK antibody test result (in those with external result)

		External antibody testing		
		Negative	Positive	Total
KCL-CHECK antibody test result	Negative	103	14	117
	Positive	2	19	21 (15%)
	Total	105	33 (24%)	138

2x2 stats	
agreement	88%
(chance agreement)	68%)
kappa	0.636
sensitivity	58%
specificity	98%
Positive predictive value	90%
Negative predictive value	88%

Sensitivity, specificity, PPV and NPV refer to the ability of the KCL-CHECK antibody test to detect those who have tested positive on an external antibody test

Appendix 1:

STROBE Statement—Checklist of items that should be included in reports of ***cohort studies***

	Item No	Recommendation	Section
**Title and abstract**	1	(*a*) Indicate the study's design with a commonly used term in the title or the abstract	Title
		(*b*) Provide in the abstract an informative and balanced summary of what was done and what was found	Abstract
**Introduction**			
Background/rationale	2	Explain the scientific background and rationale for the investigation being reported	1
Objectives	3	State specific objectives, including any prespecified hypotheses	1 (descriptive)
**Methods**			
Study design	4	Present key elements of study design early in the paper	2.1-2.2
Setting	5	Describe the setting, locations, and relevant dates, including periods of recruitment, exposure, follow-up, and data collection	2.1 (and separate protocol)
Participants	6	(*a*) Give the eligibility criteria, and the sources and methods of selection of participants. Describe methods of follow-up	2.1 (and separate protocol)
		(*b*) For matched studies, give matching criteria and number of exposed and unexposed	n/a
Variables	7	Clearly define all outcomes, exposures, predictors, potential confounders, and effect modifiers. Give diagnostic criteria, if applicable	2.2
Data sources/ measurement	8*	For each variable of interest, give sources of data and details of methods of assessment (measurement). Describe comparability of assessment methods if there is more than one group	2.2
Bias	9	Describe any efforts to address potential sources of bias	n/a
Study size	10	Explain how the study size was arrived at	n/a
Quantitative variables	11	Explain how quantitative variables were handled in the analyses. If applicable, describe which groupings were chosen and why	2.2-2.3
Statistical methods	12	(*a*) Describe all statistical methods, including those used to control for confounding	2.3
		(*b*) Describe any methods used to examine subgroups and interactions	n/a
		(*c*) Explain how missing data were addressed	2.3-3.1
		(*d*) If applicable, explain how loss to follow-up was addressed	3.1
		(*e*) Describe any sensitivity analyses	2.3-3.1
**Results**			
Participants	13*	(a) Report numbers of individuals at each stage of study—eg numbers potentially eligible, examined for eligibility, confirmed	Figure 1 / Table ST1

		eligible, included in the study, completing follow-up, and analysed	
		(b) Give reasons for non-participation at each stage	Figure 1
		(c) Consider use of a flow diagram	Figure 1
Descriptive data	14*	(a) Give characteristics of study participants (eg demographic, clinical, social) and information on exposures and potential confounders	Tables ST1-3
		(b) Indicate number of participants with missing data for each variable of interest	Table ST1, section 3.1
		(c) Summarise follow-up time (eg, average and total amount)	Section 3.1
Outcome data	15*	Report numbers of outcome events or summary measures over time	Table 2
Main results	16	(*a*) Give unadjusted estimates and, if applicable, confounder-adjusted estimates and their precision (eg, 95% confidence interval). Make clear which confounders were adjusted for and why they were included	3.2
		(*b*) Report category boundaries when continuous variables were categorized	2.2
		(*c*) If relevant, consider translating estimates of relative risk into absolute risk for a meaningful time period	n/a
Other analyses	17	Report other analyses done—eg analyses of subgroups and interactions, and sensitivity analyses	3.1
**Discussion**			
Key results	18	Summarise key results with reference to study objectives	4
Limitations	19	Discuss limitations of the study, taking into account sources of potential bias or imprecision. Discuss both direction and magnitude of any potential bias	4
Interpretation	20	Give a cautious overall interpretation of results considering objectives, limitations, multiplicity of analyses, results from similar studies, and other relevant evidence	4
Generalisability	21	Discuss the generalisability (external validity) of the study results	4
**Other information**			
Funding	22	Give the source of funding and the role of the funders for the present study and, if applicable, for the original study on which the present article is based	Funding[a]

Table note (a): KCL-CHECK is funded by King's College London. All investigators are employed by King's College London. Two of the principal investigators are also managers in King's College London. Decisions regarding study design, analysis, interpretation and decision to publish have been taken by the named investigators without external influence.

*Give information separately for exposed and unexposed groups.

Note: An Explanation and Elaboration article discusses each checklist item and gives methodological background and published examples of transparent reporting. The STROBE checklist is best used in conjunction with this article (freely available on the Web sites of PLoS Medicine at http://www.plosmedicine.org/, Annals of Internal Medicine at http://www.annals.org/, and Epidemiology at http://www.epidem.com/). Information on the STROBE Initiative is available at http://www.strobe-statement.org.

Appendix 2: PDF of instructions sent to participants for home testing of antibodies

Dear [FirstName],

Thank you for your participation in the KCL CHECK (Coronavirus: Health & Experiences of Colleagues at King's) study. You are contributing to research concerning an unprecedented situation and are helping to improve understanding of how the COVID-19 (Coronavirus) pandemic is affecting the work, health and wellbeing of staff and postgraduate research students at King's College London.

As part of KCL CHECK, you consented to taking part in the virology component of the study and we are pleased to enclose your antibody home-testing kit. This test is comprised of a simple device used to detect the presence or absence of two types of COVID-19 antibodies (IgG and IgM) in a blood sample. Studies have shown the body produces these antibodies after experiencing COVID-19 infection. However, this is a research study and not a clinical test, so you should not rely only on the result of this test as either confirming that you have not had COVID-19 if the test is negative, or that you have had COVID-19 if the test is positive.

The test involves pricking your fingertip and taking a small blood sample which will be analysed in the test cassette supplied. This process should take around 10 minutes. You will need to photograph the result within 10-15 minutes of completing the procedure and upload the picture to the KCL CHECK website: www.kcl-check.org/test-results.

Enclosed you will find:

1. A Coronavirus Rapid Test Cassette and kit (contents explained further in the instructions);
2. Step-by-step instructions for how to perform the test, how to interpret the result, and how to share the result with the KCL CHECK Research Team. **Please read these before conducting the test and follow the steps carefully.**

You can find more information about the KCL CHECK study on the Participant Information Sheet at www.kcl-check.org/pis. The FAQ section of our website can also be found here: www.kcl-check.org/faq. If you have any further questions or if you would like to speak to the KCL CHECK Research Team, please email check@kcl.ac.uk.

Thank you for participating in this important research.

Best wishes,
KCL CHECK Research Team

Please turn over for the step-by-step instructions when you are ready to begin the test

KCL CHECK
Instructions for COVID-19 antibody home-testing kit
(COVID-19 IgG/IgM Rapid Test Cassette)

Test kit contents:

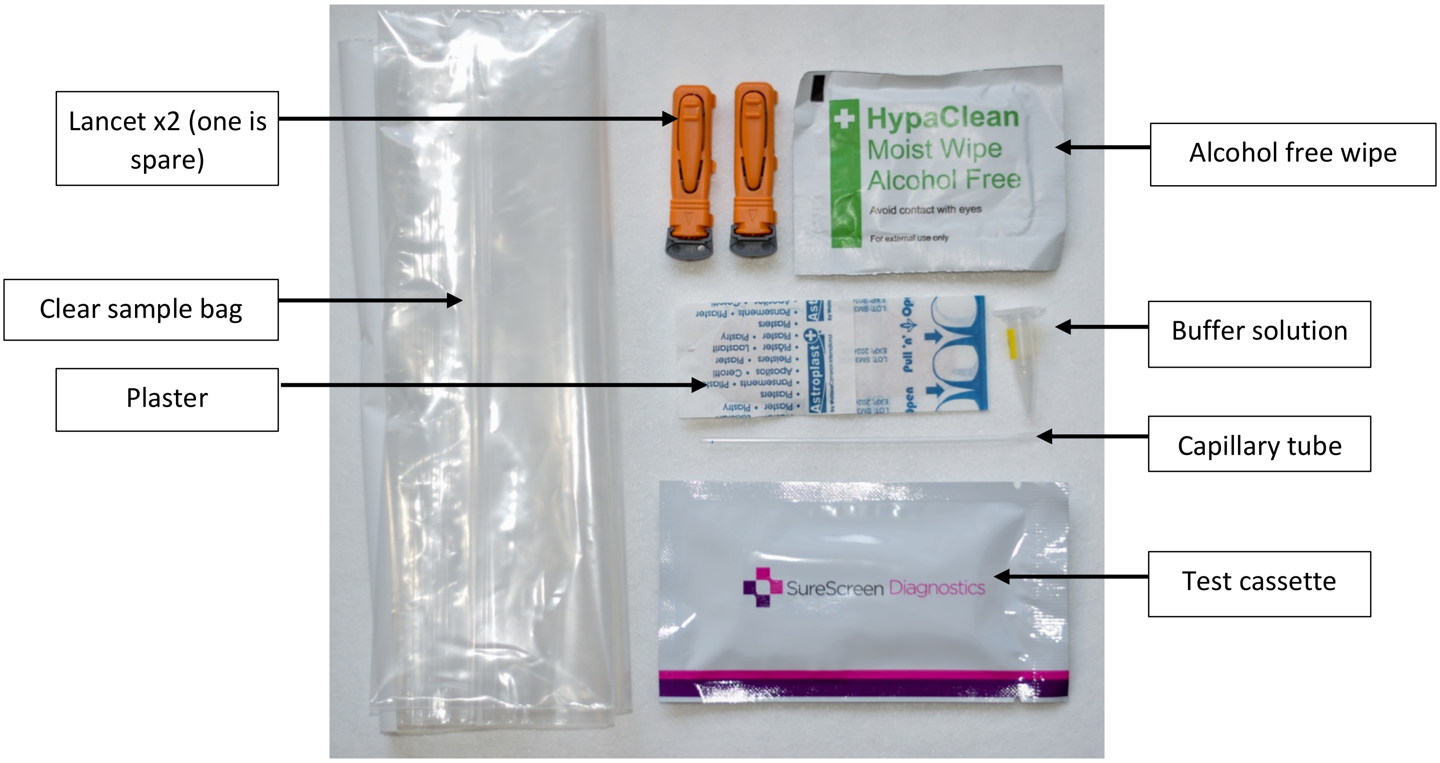

You also need a **clean tissue**, a **timer** and a way **to photograph** your result.

Step-by-step instructions

The test must be interpreted and photographed within 10-15 minutes of completing the procedure to work correctly, so make sure you have time before starting.

Step 1: Set-up

Before you begin, check you have everything you need (use the contents image above for reference). Then, remove the test cassette from the packaging **(use within 1 hour of opening)** and lay out the contents of your test kit on a clean, level surface.

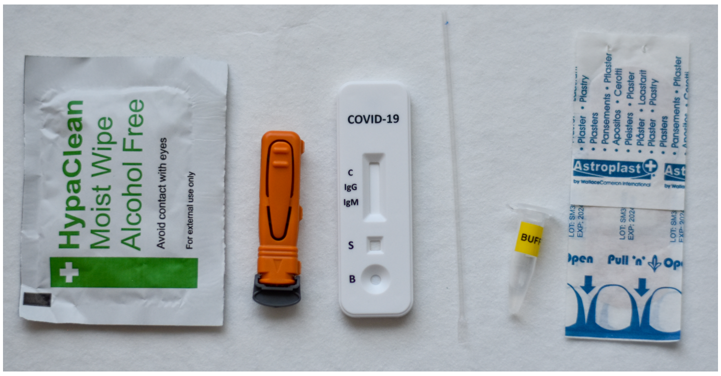

Step 2: Wash your hands and wipe your fingertip

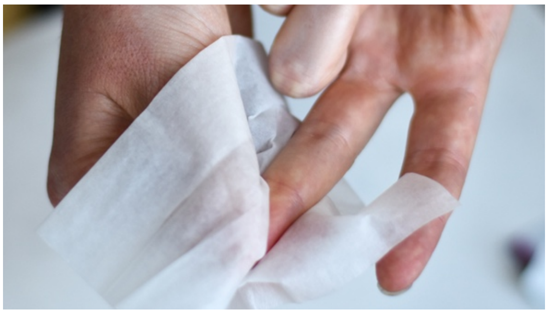

Using warm water, wash your hands. This helps with blood flow. Then, select either your middle or ring finger, and use the alcohol-free wipe to clean it.

Step 3: Puncture the skin using a lancet

To use the lancet, **twist** the grey cap clockwise, but **do not pull it**. As you twist, the cap will release on its own. Use the second lancet if you have problems with the first.

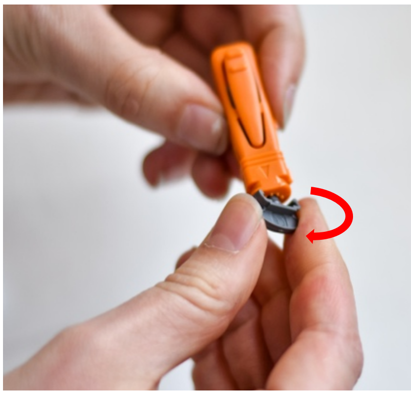

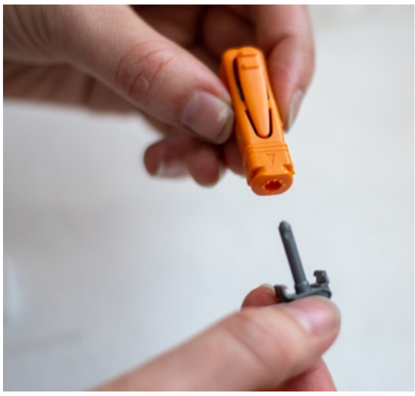

With the cap removed, massage your hand without touching the puncture site by rubbing down the hand towards the fingertip to encourage blood flow.

Then, hold the lancet firmly on your fingertip, and press the button to release the needle.

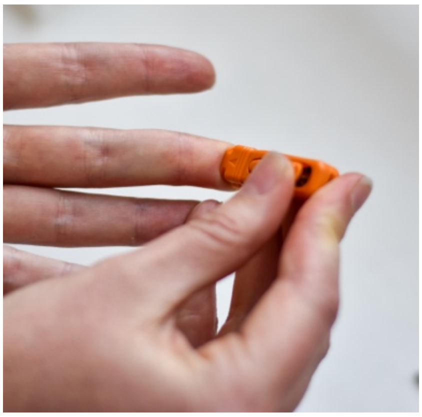

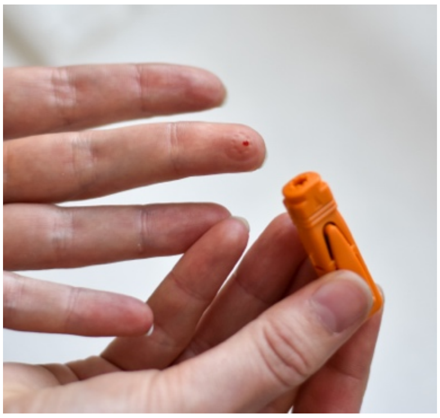

Wipe away the first sign of blood with a clean tissue.

Step 4: Add a drop of blood to the test cassette

Gently run your hand from the wrist up to the finger to form a rounded drop of blood. Release one drop of blood into the specimen well of the test cassette, **<u>this is marked 'S'</u>**.

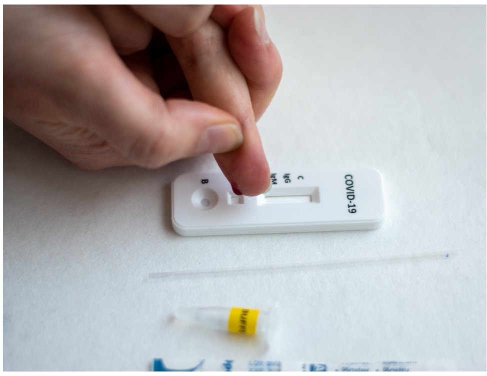

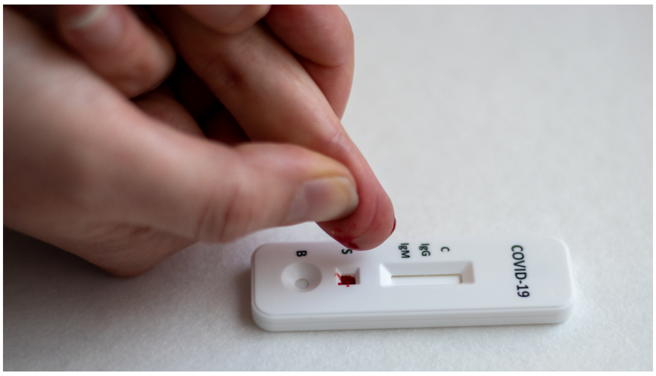

Step 5: Add the buffer to the test cassette

Open the buffer tube and add the buffer to the cassette using the capillary tube. To use the capillary, press firmly half-way down, then place the open end into the buffer and release. This will draw up the liquid into the capillary tube.

Then, drop the buffer solution onto the test cassette area **<u>marked 'B'</u>** by squeezing the capillary tube. You must **<u>use all the buffer</u>** in the buffer tube, so you may have to repeat this process, try to do it as quickly as possible.

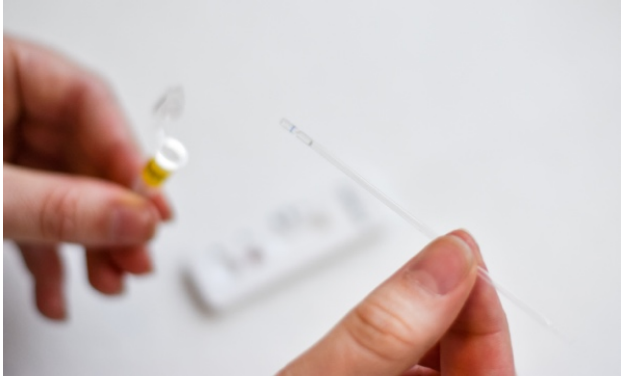

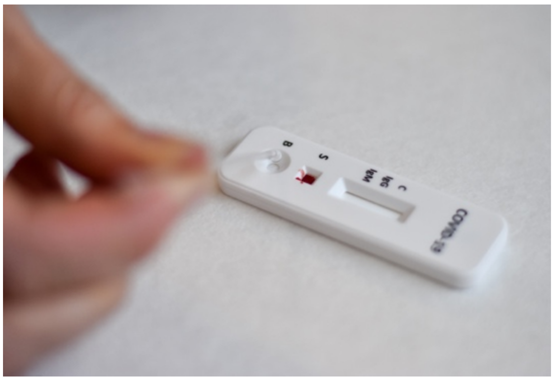

Step 6: Start the timer and record the current time

As soon as you have added the buffer, start a timer for **10 minutes**. Check the current time and record it in the box on the next page as the 'test start time'. Please record the time in a 24-hour format (e.g.16:55).

While you wait, use the plaster to seal the puncture site on your fingertip.

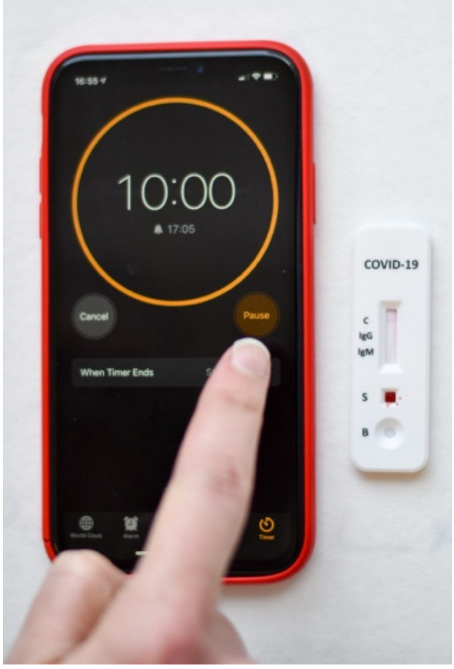

Step 7: Check your result

After **10 minutes**, coloured line(s) should appear on the test cassette. You can use the guide below to check your results.

Result Interpretation Guide: (See Figure 1)

Note: The COVID-19 IgG/IgM Rapid Test Cassette will only indicate the presence of COVID-19 antibodies and should not be used as the <u>sole</u> criteria for the diagnosis of COVID-19. A negative result does not at <u>any time</u> exclude the possibility of COVID-19 infection. Similarly, a positive result <u>does not</u> confirm a previous COVID-19 infection. Please follow current NHS guidelines regarding COVID-19 symptoms and diagnosis.

IgG and IgM POSITIVE: Three lines appear. A colored line appears each in the control region (C), the IgG test region and IgM test region. The result is positive for IgG & IgM antibodies and may indicate an immune response to a COVID-19 infection.

IgG POSITIVE: Two lines appear. A colored line appears in the control region (C), and also in the IgG test region. The result may indicate the presence of the specific antibody produced by the immune system in response to a COVID-19 infection.

IgM POSITIVE: Two lines appear. A colored line appears in the control region (C), and also in the IgM test region. The result may indicate the presence of the general antibody produced by the immune system as a first response to a COVID-19 infection.

NEGATIVE: One colored line appears in the control region (C). No lines appear in the IgG and IgM test regions. This may indicate no immune response present for a COVID-19 infection

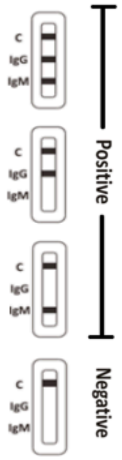

INVALID: Control line fails to appear. This could happen for multiple reasons. Please ensure that you still record and share the results so that the research team are aware.

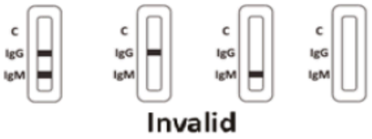

Figure 1: Result Interpretation

Step 8: Record your result

Without delay, place your test cassette in the box below and record your result, as well as the current time in ‘test end time’ (this should be in a 24-hour format, e.g. 17:05).

Place test cassette here	Please complete the following based on your interpretation of the result:<br><br>Positive ☐<br><br>Negative ☐<br><br>Invalid ☐<br><br>Test start time: ____:____<br><br>Test end time: ____:____<br><br>1|1|1

Take a photograph of the result. Make sure that the whole box is visible, and that the image is clear (see examples below). This must be done **within 10-15 minutes** of you completing the procedure to generate a valid result – **do not check or photograph the result after 20 minutes.**

✓ The whole box is clearly visible, with all information complete.

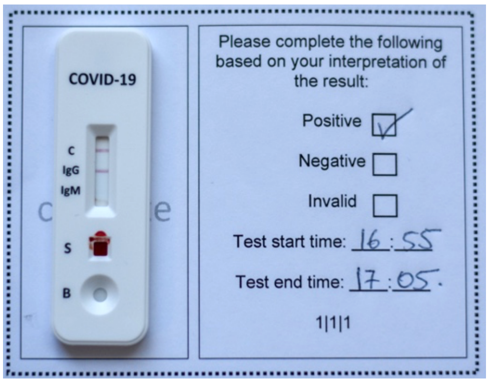

✗ Test obstructing information.

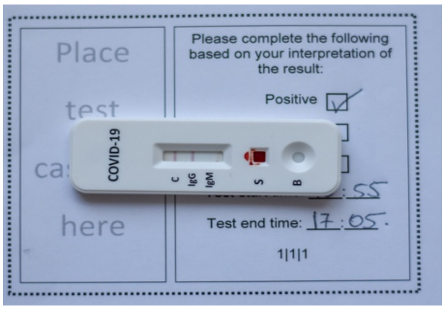

Whole box not visible.

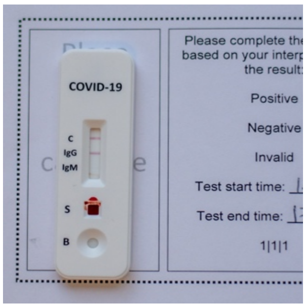

Image is blurry.

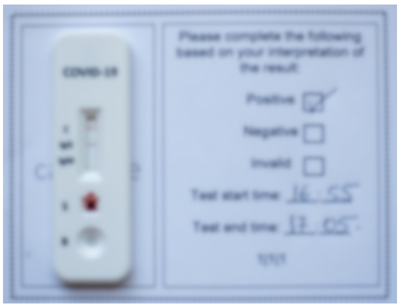

Step 9: Upload your photograph

Visit **www.kcl-check.org/test-results** via your web browser and upload and submit your photograph to share the test result with the KCL CHECK research team.

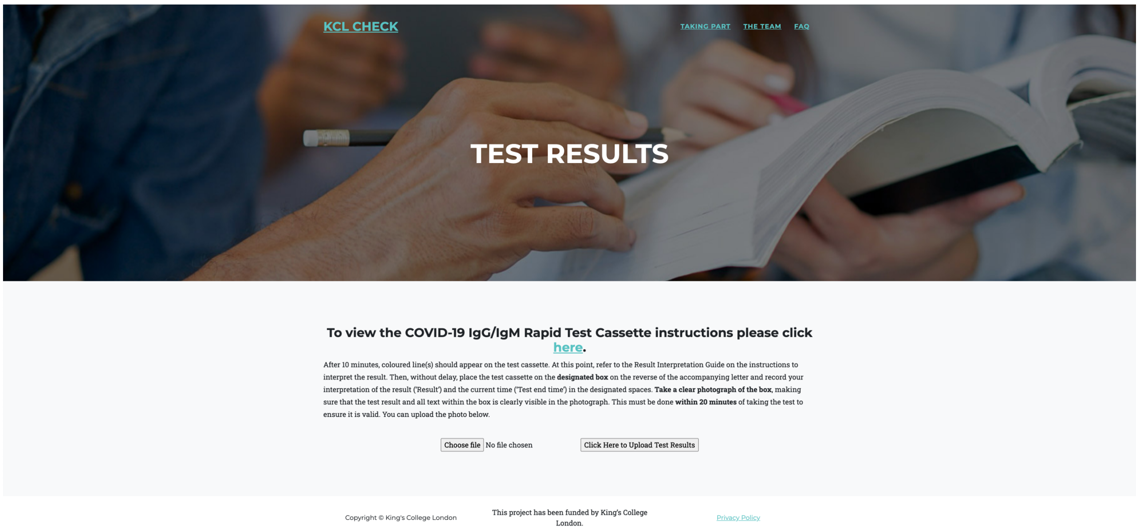

Step 10: Dispose of the kit in the clear sample bag

Place the used test cassette, buffer, lancet, capillary tube and alcohol wipe into the clear sample bag. **Seal the bag and dispose of in general waste.**
